# A Multilevel Integrated Intervention to Reduce the Impact of HIV Stigma on HIV Treatment Outcomes Among Adolescents Living With HIV in Uganda: Protocol for a Randomized Controlled Trial

**DOI:** 10.2196/40101

**Published:** 2022-10-05

**Authors:** Massy Mutumba, Fred Ssewamala, Rashida Namirembe, Ozge Sensoy Bahar, Proscovia Nabunya, Torsten Neilands, Yesim Tozan, Flavia Namuwonge, Jennifer Nattabi, Penina Acayo Laker, Barbara Mukasa, Abel Mwebembezi

**Affiliations:** 1 Department of Health Behavior & Biological Sciences School of Nursing University of Michigan Ann Arbor, MI United States; 2 Brown School Washington University in St Louis St Louis, MO United States; 3 International Center for Child Health and Development Masaka Uganda; 4 Division of Prevention Science Department of Medicine University of California, San Francisco San Francisco, CA United States; 5 Sam Fox School of Design and Visual Arts Washington University in St Louis St Louis, MO United States; 6 Mildmay Uganda Kampala Uganda; 7 Reach the Youth Uganda Kampala Uganda

**Keywords:** HIV/AIDS, stigma, adolescents, school

## Abstract

**Background:**

HIV stigma remains a formidable barrier to HIV treatment adherence among school-attending adolescents living with HIV, owing to high levels of HIV stigma within schools, rigid school structures and routines, lack of adherence support, and food insecurity. Thus, this protocol paper presents an evidence-informed multilevel intervention that will simultaneously address family- and school-related barriers to HIV treatment adherence and care engagement among adolescents living with HIV attending boarding schools in Uganda.

**Objective:**

The proposed intervention—Multilevel Suubi (MSuubi)—has the following objectives: examine the impact of M-Suubi on HIV viral suppression (primary outcome) and adherence to HIV treatment, including keeping appointments, pharmacy refills, pill counts, and retention in care; examine the effect of M-Suubi on HIV stigma (internalized, anticipated, and enacted), with secondary analyses to explore hypothesized mechanisms of change (eg, depression) and intervention mediation; assess the cost and cost-effectiveness of each intervention condition; and qualitatively examine participants’ experiences with HIV stigma, HIV treatment adherence, and intervention and educators’ attitudes toward adolescents living with HIV and experiences with group-based HIV stigma reduction for educators, and program or policy implementation after training.

**Methods:**

MSuubi is a 5-year multilevel mixed methods randomized controlled trial targeting adolescents living with HIV aged 10 to 17 years enrolled in a primary or secondary school with a boarding section. This longitudinal study will use a 3-arm cluster randomized design across 42 HIV clinics in southwestern Uganda. Participants will be randomized at the clinic level to 1 of the 3 study conditions (n=14 schools; n=280 students per study arm). These include the bolstered usual care (consisting of the literature on antiretroviral therapy adherence promotion and stigma reduction), multiple family groups for HIV stigma reduction plus family economic empowerment (MFG-HIVSR plus FEE), and Group-based HIV stigma reduction for educators (GED-HIVSR). Adolescents randomized to the GED-HIVSR treatment arm will also receive the MFG-HIVSR plus FEE treatment. MSuubi will be provided for 20 months, with assessments at baseline and 12, 24, and 36 months.

**Results:**

This study was funded in September 2021. Participant screening and recruitment began in April 2022, with 158 dyads enrolled as of May 2022. Dissemination of the main study findings is anticipated in 2025.

**Conclusions:**

MSuubi will assess the effects of a combined intervention (family-based economic empowerment, financial literacy education, and school-based HIV stigma) on HIV stigma among adolescents living with HIV in Uganda. The results will expand our understanding of effective intervention strategies for reducing stigma among HIV-infected and noninfected populations in Uganda and improving HIV treatment outcomes among adolescents living with HIV in sub-Saharan Africa.

**Trial Registration:**

ClinicalTrials.gov NCT05307250; https://clinicaltrials.gov/ct2/show/NCT05307250

**International Registered Report Identifier (IRRID):**

PRR1-10.2196/40101

## Introduction

### Background

HIV/AIDS among adolescents remains a public health concern worldwide. Over 1.7 million children aged<15 years live with HIV [1], and almost half of all new HIV infections worldwide occurs in youth aged 15 to 24 years [2]. Sub-Saharan Africa (SSA) bears the brunt of the HIV epidemic in children and adolescents, accounting for more than 88% of the global population of adolescents living with HIV and 80% of the 460,000 new infections worldwide among adolescents [1]. Uganda is home to more than 170,000 adolescents living with HIV. This figure is expected to increase as adolescents remain highly vulnerable to HIV infection [3], perinatal transmission of HIV continues to occur [4], and expanded access to antiretroviral therapy (ART) increases the longevity of persons infected with HIV [5-7]. However, similar to other countries [8-11], adolescents living with HIV in Uganda have lower levels of ART adherence (<50%) [12,13], low rates of viral suppression [14], and high attrition from HIV care than children and adults living with HIV [15-18]. Nonadherence to HIV care potentiates secondary transmission of drug-resistant HIV among nonvirally suppressed adolescents living with HIV engaging in unprotected sex [19-21], and undermines global efforts to eradicate AIDS [22]. Without improvements in HIV prevention, testing, and treatment, a staggering 360,000 adolescents may die of AIDS-related diseases by 2030 [4].

Adolescents living with HIV in boarding schools are more disadvantaged and have lower levels of HIV treatment adherence. HIV stigma [23-25], poverty (including food insecurity) [26,27], and poor mental health [13,28-31] are increasingly being listed as the most potent barriers to ART adherence in Uganda and SSA. The school social context is very disadvantageous for adolescents living with HIV. First, adolescents living with HIV lack the family support that typically facilitates treatment adherence [13,32]. Second, the lack of privacy (given the living arrangements) coupled with high levels of HIV stigma (internalized, anticipated, and enacted) heightens adolescents living with HIV’s concerns about unintentional disclosure of HIV status. In our preliminary studies, adolescents living with HIV reported shaming, peer rejection, and exclusion from school activities after disclosure of their HIV status, resulting in suicidal thoughts and thoughts of school dropout [33,34]. Third, poverty-related food insecurity, manifesting as lack of food to accompany medications, is another barrier [35-38]. Adolescents living with HIV are often advised to take their drugs at bedtime to reduce medication side effects (eg, drowsiness or nausea) that may interfere with school activities. However, taking drugs on an empty stomach usually amplifies side effects. Poor parents are often unable to supplement their children’s school meals to support treatment adherence. The aim of this study is to examine the effects of an evidence-informed multilevel intervention—Multilevel Suubi (M-Suubi)—that seeks to simultaneously address multiple barriers to HIV treatment adherence and care engagement among school-attending adolescents living with HIV in Uganda.

High levels of HIV stigma persist in SSA, including Uganda [39,40], creating a formidable barrier to HIV treatment adherence among adolescents living with HIV [23-25]. Stigma is a societal process that manifests at multiple socioecological levels [41,42]. HIV stigma can manifest internally (ie, internalized and anticipated stigma) based on the perceived negative public attitude and encompassing feelings of one as reprehensible, damaged, and ineffective. These feelings may lead to mental health problems such as depression, posttraumatic stress disorder, suicidal ideation [33,43-45], feelings of loneliness and social isolation [23,46,47], diminished physical health [48-51], sexual risk behavior [52,53] and poor treatment adherence [20,25,54-57]. Adolescents living with HIV experience internal and external HIV stigma (ie, anticipated and enacted stigma, respectively) within homes and schools [58]—the most important adolescent development contexts. Many adolescents living with HIV live in extended family settings (owing to orphanhood), where enacted stigma is perpetrated through rejection, verbal insults, and ostracism [23,59-63]. Family members are often condemned and stigmatized in similar ways because of their association with adolescents living with HIV (ie, associative stigma) [64], which can negatively affect family functioning. Within schools, HIV stigma is rampant among peers and educators (eg, teachers, administrators, and nurses), manifesting as gossip, rejection, harassment, social isolation, and loss of friendship and social support [23,58,60,65]. Educators are often indiscreet, ignorant about HIV/AIDS, and uncaring and unresponsive to enacted stigma within schools [65]. These experiences can diminish adolescents living with HIV’s ability to develop a positive self-concept and form strong bonds with family members and peers and increase their risk of mental health problems. HIV stigma and social exclusion lead to, or exacerbate mental health symptoms (eg, depression and suicidal ideation) and contribute to school dropout [23,33,34,58,60,66]. HIV stigma also undermines HIV treatment adherence and impedes adolescents living with HIV’s access to social support in school settings [65,67]. These adverse effects of HIV underscore the urgent need for interventions to reduce HIV stigma within schools and families.

HIV stigma exists at the intersection between HIV and poverty and perpetuates disparities among people living with HIV by concentrating the adverse impacts of HIV stigma on the poor [68,69]. Poverty is rampant among HIV-affected households [70-72] and is a significant risk factor for HIV acquisition [73] and poor HIV treatment outcomes [26,27]. People living with HIV from poverty-stricken households face greater challenges in accessing and sustaining HIV treatment owing to economic challenges, such as lack of transport to clinics [74,75] and inadequate meals to support medication adherence [36,38,76]. Numerous studies conducted in SSA [35-38], have identified food insecurity as a formidable barrier to ART adherence. Within boarding schools, inadequate nutrition or lack of foods or snacks may dissuade adolescents living with HIV from taking their medications because of concerns that taking drugs on an empty stomach can intensify side effects. Poverty can adversely affect the quality of family relationships, including parent-child communication, involvement [77-79] and parenting skills [80,81], which increases adolescents’ susceptibility to poor outcomes such as emotional and behavioral adjustment [78,82-86].

In SSA, where HIV has disrupted the social function of the family, schools are potential substitutes for providing supportive developmental contexts that can mitigate the risks for poor outcomes in vulnerable children, including adolescents living with HIV [87,88]. For adolescents living with HIV, the typical developmental challenges of adolescence are compounded by HIV-related challenges such as managing complex drug regimens, coping with multiple bereavements, comorbidities, and social challenges (eg, HIV disclosure) [89-91]. As such, adolescents living with HIV need additional support to successfully negotiate adolescence. However, poverty and food insecurity undermine their ability to fully participate in school, and HIV stigma in schools undermines their potential to support adolescents living with HIV. School-attending adolescents spend a large part of the day away from home, and for adolescents living with HIV, this means that they must take their daily medication while at school. Treatment is even more challenging for more than 60% of adolescents living with HIV who spend 9 to 10 months a year away from home in boarding sections—a form of parental opt-in institutional care with limited family visitation (typically monthly). Adolescents living with HIV in boarding schools are vulnerable to HIV stigma, abuse, poor nutrition, mental and physical difficulties, and poverty [23,58-60,67] and have significantly lower levels of ART adherence compared with adolescents living with HIV in day schools [92]. The lack of attention to addressing the school-related needs of the large population of in-school adolescents living with HIV in Uganda and other high HIV burden countries in SSA has adverse consequences for ongoing efforts to end the AIDS epidemic [22]. Targeting HIV stigma within schools is necessary to enhance HIV treatment outcomes and the educational achievement for adolescents living with HIV in SSA.

Recent systematic reviews indicate that interventions to reduce HIV stigma among adolescents living with HIV in resource-limited settings are almost nonexistent [93-98]. For example, of the 48 stigma reduction interventions [97], only 3 studies were aimed at people living with HIV in SSA, and none of these interventions targeted adolescents living with HIV or assessed the impact of stigma reduction on HIV treatment outcomes among adolescents living with HIV. Moreover, these interventions tend to be single-level focused (eg, focus exclusively on family) and use a limited range of intervention strategies [97]. Although several interventions have shown promise in improving HIV treatment adherence among adolescents living with HIV [99-102], they mostly focus on adolescents living with HIV commuting daily from home. However, the majority (>60%) of school-going children in Uganda (and in many sub-Saharan African countries heavily impacted by HIV) spend their time in boarding sections. The lack of attention to this group undermines the efforts to achieve the 95-95-95 targets in SSA. Building on our experience using multiple family groups (MFGs) and family economic empowerment (FEE) interventions to improve health outcomes among adolescents recruited from schools and clinics [26,78,103-106] and supported by the literature on the impact of HIV stigma within families and schools [23,33,34,58,60,65-67] and the impact of FEE on HIV treatment outcomes [107-111], we propose testing a culturally acceptable asset-based multilevel intervention (M-Suubi) that targets HIV stigma within schools and families to improve HIV treatment outcomes among adolescents living with HIV.

### Objectives

Although several interventions have shown promise in improving HIV treatment adherence among adolescents living with HIV [99-102], they mostly focus on adolescents living with HIV commuting daily from home. However, most school-going children in Uganda and many sub-Saharan African countries heavily impacted by HIV spend their time in boarding sections. The lack of attention to this group undermines the efforts to achieve the 95-95-95 targets in SSA. Our research finds that MFG and FEE [27,100] can improve HIV care outcomes among adolescents living with HIV. Moreover, consistent with the existing literature in SSA [33], our recent combination intervention study, Bridges, situated within Ugandan schools, points to the importance of building supportive familial and school environments for adolescents affected by HIV/AIDS, including adolescents living with HIV [112-115]. Building on prior experience and evidence of effective HIV stigma reduction strategies [40,95,98,116], we propose to examine an evidence-informed multilevel intervention called M-Suubi (the word *suubi* means hope) intervention that seeks to simultaneously address multiple barriers to HIV treatment adherence and care engagement among adolescents living with HIV attending boarding schools in Uganda. M-Suubi comprises of three study conditions: (1) Bolstered usual care consisting of literature on ART adherence promotion and stigma reduction, (2) MFG for HIV stigma reduction plus FEE (MFG-HIVSR plus FEE), and (3) group-based HIV stigma reduction for educators (GED-HIVSR). The study is guided by the HIV stigma framework [64], asset theory [117,118], and family system theory [119,120] and has the following goals:

- Aim 1: examine the impact of M-Suubi on HIV viral suppression (primary outcome) and adherence to HIV treatment, including keeping appointments, pharmacy refills, pill counts, and retention in care.
- Aim 2: examine the effect of M-Suubi on HIV stigma (internalized, anticipated, and enacted), with secondary analyses to explore hypothesized mechanisms of change (eg, depression) and intervention mediation.
- Aim 3: assess the cost and cost-effectiveness of each intervention condition.
- Aim 4: qualitatively examine participants’ experiences with HIV stigma, HIV treatment adherence, and intervention and educators’ attitudes toward adolescents living with HIV and experiences with GED-HIVSR and program/policy implementation after training.

## Methods

### Study Overview

**Figure 1:**
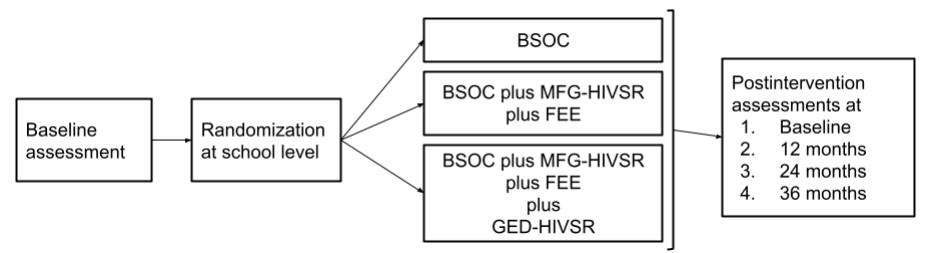
Study conditions and assessments. BSOC: bolstered standard of care; FEE: family economic empowerment; GED-HIVSR: group-based HIV stigma reduction for educators; MFG-HIVSR: multiple family groups HIV stigma reduction for educators.

M-Suubi is a 5-year multilevel mixed methods randomized controlled trial. As shown in Figure 1, the M-Suubi intervention will be evaluated using a 3-arm cluster randomized trial implemented across 42 community health centers (with HIV clinics), targeting adolescents living with HIV aged 10 to 17 years and attending primary and secondary schools with a boarding section (n=14 clinics per arm; n=280 students per study arm). Adolescents living with HIV will be randomized at the clinic level to one of the three study conditions: (1) bolstered usual care, (2) MFG-HIVSR plus FEE, and (3) MFG-HIVSR plus FEE plus GED-HIVSR (Figure 1). M-Suubi will be provided for 20 months, with assessments at baseline and 12, 24, and 36 months.

### Theoretical Framework

This proposal is guided by the HIV stigma framework [64], asset theory [117,118], and family systems theory [119,120]. The HIV stigma framework [64] suggests that HIV stigma affects people living with HIV via three distinct mechanisms: stereotyping (cognitive), prejudice (affective), and discrimination (behavioral). M-Suubi focuses on all forms of HIV stigma (internalized, anticipated, and enacted) and uses a range of strategies (eg, education, skill building, empowerment, and empathy) to address HIV stigma at the individual, interpersonal, and institutional levels [95-98]. Consistent with a multilevel approach to HIV stigma reduction, M-Suubi targets following three ecological levels: (1) school using GED-HIVSR, (2) family using MFG-HIVSR plus FEE, and (3) individual (adolescents living with HIV) using locally adapted Suubi-MAKA [105,121,122] and Suubi+Adherence [26,123,124] curricula. All intervention arms use a variety of strategies (eg, education, cognitive restructuring, empowerment, and skill building) to address HIV stigma**.**

Our rationale for pairing MFG-HIVSR with FEE comes from mounting evidence that cognitive and behavioral changes in adolescents are influenced by economic stability, whereas family support and protective processes are needed to reinforce and maintain engagement in protective health behaviors. MFG-HIVSR will provide a safe setting for parents and their children to address HIV stigma, foster family communication, facilitate optimism and morale by normalizing shared experiences with other families, and enhance interpersonal and coping skills. FEE will alleviate the impact of family economic insecurity; hence, mitigating the potential impact of food insecurity on ART adherence and caregiver engagement among the study participants. More specifically, financial security will enable parents to support their children in schools through visitations and supplemental nutrition. For adolescents living with HIV, internalized stigma is targeted in the MFG-HIVSR using the locally adapted Suubi+Adherence curriculum [123] that discusses several adherence barriers including HIV stigma. These strategies will impact a range of psychological, behavioral, and health outcomes among adolescents living with HIV, caregivers, and educators. Guided by the HIV stigma framework, the GED-HIVSR targets educators (ie, teachers, school nurses, matrons, and administrators) to build HIV knowledge, foster empathy, and build support for adolescents living with HIV in boarding schools, whereas the MFG-HIVSR targets HIV stigma within families. Asset-based and family systems theory guides the MFG-HIVSR and FEE to alleviate poverty within families. Asset theory also guides our approach to GED-HIVSR, where we draw on skills and values of educators, emphasize the identification of resources within schools and the local community, and encourage educators to develop their own plans to support the needs of adolescents living with HIV, potentially promoting the ownership of intervention activities.

### Setting and Study Population

The target populations for this study are adolescents living with HIV, their caregivers, and educators within the Greater Masaka region in southwestern Uganda, a region heavily affected by HIV [125]. We plan to recruit 840 adolescents living with HIV and their caregivers from 42 community health centers (with HIV clinics) and their primary and secondary schools. We will work with clinics affiliated with Reach The Youth, our local implementing partner. For adolescents living with HIV randomized to treatment arm 2 (ie, MFG-HIVSR plus FEE and GED-HIVSR), we will include all the schools in the GED-HIVSR component, irrespective of the number of participants attending the school. From each of the selected schools, we will recruit up to five educators, including school nurses, head administrators, and teachers.

### Inclusion and Exclusion Criteria

The following are the inclusion criteria for participants: (1) the individual is HIV positive, defined as an adolescent who has tested positive with confirmation by medical report and has been disclosed to; (2) the individual is prescribed ART; (3) the individual is living within a family (defined broadly, not necessarily with biological parents); and (4) the individual is aged 10 to 17 years and enrolled in a primary or secondary school with a boarding section within the Greater Masaka region. At the clinic level, all eligible adolescents living with HIV from a particular household will be enrolled in the study and assigned to the same study condition.

The family inclusion criterion is that the participants must be caregivers of adolescents living with HIV who agree to participate in the study.

The educator inclusion criterion is that the participants must be teachers, school nurses, and administrators in the target schools who agree to participate in the study. For adolescents living with HIV randomized to treatment arm 2 (ie, MFG-HIVSR plus FEE and GED-HIVSR), we will include all the schools in the GED-HIVSR component, irrespective of the number of participants attending the school. All educators will be required to consent to participate in the study individually.

The following are the exclusion criteria: (1) significant cognitive impairment that interferes with the participants’ understanding of the informed consent process or (2) inability/unwillingness to commit to completing the study.

### Enrollment

After identifying potential study participants, we will compile a list of secondary schools attended, including the number of potential participants in each school, associated school features (ie, location and size), and willingness of these schools to participate in the study. Clinics will be randomized to 1 of 3 study arms, and all adolescents living with HIV and their caregivers will be enrolled in the study arm associated with their clinic. Only adolescents and their caregivers who meet inclusion criteria will be recruited. To characterize any potential bias in enrollment, we will collect information about the clinics (eg, location, clinic size, and reasons for nonparticipation) and use HIV clinic information (eg, sociodemographics and viral suppression rates) to characterize the potential bias from adolescents living with HIV and clinics that decline to participate in the study. For participants randomized to the GED-HIVSR intervention arm, we will collect information on the school location, type (eg, private or government-supported), size, and reasons for nonparticipation.

### Intervention Conditions

#### Control Arm: Bolstered Usual Care

All participants (in the control and treatment arms) will receive medical and psychosocial support as part of the bolstered usual care. All public clinics, including our study sites, follow procedures for pediatric ART initiation and monitoring as outlined in the National Guidelines for pediatric HIV care in Uganda [126]. As part of medical care, ART is prescribed by physicians and dispensed monthly by a pharmacist at the clinic. Specifically, immediately after initiation, or if clinically unstable, adolescents living with HIV are seen more frequently (weekly to monthly). Laboratory data—viral load (VL) and CD4 counts—are collected every 6 months until the patient is stabilized and then checked annually. For M-Suubi, data regarding HIV viral load, pharmacy refills, and pill counts will be collected from the charts. Psychosocial care is primarily provided by lay counselors trained in standardized ART adherence counseling. Typically, each patient receives 2 to 4 sessions of adherence counseling at initiation and when nonadherence is identified. Lay counselors also assist families with other psychosocial needs that may arise. However, adherence to counseling can vary substantially. Therefore, the usual care will be bolstered with enhanced adherence sessions to ensure more standardized and sufficient adherence counseling. All study participants will undergo 6 sessions to review HIV, ART, and ART adherence. We will bolster family communication around these topics using materials adapted from the cartoon-based curriculum used in the Suubi+Adherence study with adolescents living with HIV and their families [127]. This curriculum describes the lead characters (Mabebeere and Kamperempe), testing interactions with a nurse in which she describes the working of the HIV, ART, and adherence (including potential barriers such as HIV stigma). These materials will be discussed with the participating adolescents living with HIV to identify questions and barriers. Lay counselors in clinics have been trained to use these materials, and HIV clinics have incorporated this curriculum into their practice. Previous studies have shown that the Suubi+Adherence curriculum promotes adherence among adolescents living with HIV [127-130].

#### Treatment Arm 1: MFG-HIVSR Plus FEE

In addition to the bolstered usual care described earlier, adolescents living with HIV and their caregivers will participate in a family strengthening intervention delivered via MFG along with an FEE component. MFG is a family-centered, group-delivered, evidence-informed, strength-based 10-session (weekly) intervention for children whose families struggle with poverty and associated stressors. It integrates components of existing evidence-based practices that successfully improve parental management, mental health–promoting family processes, and family strengthening [77,104,105,121,131-134]. For the purpose of M-Suubi, MFG-HIVSR has 6 additional sessions to cover HIV stigma–related issues. The specific MFG-HIVSR session content will be based on our previous interventions [79,103-105,121,132,133,135-145]. Sessions will focus on the core MFG components, also known as 4 Rs and 2 Ss (rules, responsibility, relationships, respectful communication, stress, and social support). Sessions focused on HIV stigma will be adapted from the existing Suubi curriculum and resources from the Ministry of Health. Each session provides opportunities to contextualize the content to the realities of family life and emergent cultural and values perspectives and tailor messages to the child’s age. These will include group activities, role-plays, sharing experiences, and family take-home activities. Families (adolescents living with HIV and their caregivers) will be combined into groups of up to 10 families to promote communication and support within and among families. MFG-HIVSR sessions will last approximately 1 hour and will be delivered twice weekly during school holidays when adolescents living with HIV are more readily available. Parent peer and community health workers already trained in MFG delivery will be recruited and will receive refresher training on M-Suubi’s content on HIV stigma. During MFG-HIVSR implementation, facilitators will receive 2 hours of monthly group supervision across sites. Given the significant and protective role families play in the health and well-being of adolescents living with HIV, we expect that strengthening family functioning and dialogue by involving caregivers through MFG-HIVSR will lead to better child outcomes, including reduced HIV stigma. These services will be bolstered with an FEE component provided via a youth development account described next.

In the FEE component, adolescents living with HIV will receive a youth development account with a 1:1 matched savings program at a financial institution accredited by the Bank of Uganda. Each youth development account will be opened in the adolescent’s name, with their primary caregiver as a cosigner, until the adolescent turns 18 years, at which time a cosigner will no longer be required. This is consistent with Ugandan banking law, which prohibits children aged <18 years from independently entering into a binding contract/operating a bank account. Family members and friends of adolescents living with HIV will be allowed and encouraged to contribute to this youth development account. It will be matched at a rate of 1:1 using money from the program. The match cap (maximum amount of youth contribution to be matched by the program) will be equivalent to US $20 per month or US $480 for the 24-month intervention period. During the intervention, adolescents will have direct access to both their personal savings deposited in the youth development account and the match provided by the study to pay for food, transportation to health clinics, and other necessities that may affect adherence. Matching will not be conditioned on the usual expenditure and/or savings goals dictated by programs [133,137,146]. The unconditional design recognizes that adolescents living with HIV and attending schools face competing demands (school fees, food for medication adherence, transport to clinics, etc) and that a conditional transfer may prohibit these vital expenditures, which may have implications for antiretroviral treatment adherence. In collaboration with participating financial institutions, the youth development account will be augmented with 4 sessions of financial literacy training, covering the basic principles of financial management, saving, and asset building.

#### Treatment Arm 2: MFG-HIVSR Plus FEE Plus GED-HIVSR

In addition to the bolstered usual care and MFG plus FEE described earlier, adolescents living with HIV in this arm will receive school-level HIV stigma reduction interventions targeting teachers, school nurses, matrons, and administrators (head teachers and director of studies) in their schools. The GED-HIVSR seeks to empower educators to reduce enacted HIV stigma and build supportive structures for adolescents living with HIV within their schools. Our rationale for adding this component to our intervention package is to test the added benefit of addressing school-level HIV stigma–related challenges on HIV treatment outcomes among adolescents living with HIV. Guided by an asset-based approach and drawing on evidence-based strategies for reducing HIV stigma in non–HIV-infected populations [63,97,98,147,148] and building support for adolescents living with HIV in school settings [149-151], GED-HIVSR seeks to impart educators in the intervention schools with HIV-related knowledge, provide a safe space for educators to explore their personal values and biases that may promote or hinder their role of supportive individuals and systems for adolescents living with HIV, and empower them with knowledge and skills to act as change agents within their schools.

The GED-HIVSR will be delivered as a 2-day workshop with a booster session in years 3 to 4. The details of each topic along with the targeted domain and delivery strategies are presented in Table 1. From each intervention school, we will recruit up to five educators including the school head teacher, director of studies, and school nurse. To standardize training and provide opportunities for peer-to-peer learning through group discussions, all educators will be convened at a central location for training. Workshop content will be delivered using a range of strategies including didactic lectures, role-play, testimonials from adolescents living with HIV, digital media (ie, documentaries), and discussions/brainstorming to promote participant engagement and active learning. Workshops will establish foundational knowledge on HIV transmission and treatment and cover content on HIV stigma and its impact on adolescents living with HIV and their families. Along with testimonials from adolescents living with HIV, we will use educational documentaries that portray the marginalization of people living with HIV to highlight the perpetuation of HIV stigma and its impact on these people, including adolescents living with HIV.

**Table 1:** Topics, delivery strategies, and targeted domains of group-based HIV stigma reduction for educators.

Intervention topic			Intervention strategy		Targeted domain		Conceptual framework
HIV transmission, treatment, and prevention; misconceptions and misbeliefs about HIV; stereotypes about people living with HIV			Didactic lectures; role-play; discussions		HIV knowledge; feelings toward people living with HIV		Cognitive factors: knowledge and beliefs
HIV and AIDS stigma: understanding and defining manifestations of stigma; intersecting stigmas; consequences for adolescents living with HIV, their families and communities; awareness of HIV stigma in schools and communities; strategies for combating stigma			Educational; documentary; testimony from adolescents living with HIV		Stigma manifestations; intersecting stigma (eg, stigma and poverty); gender		Cognitive factors: knowledge and beliefs
Educators’ understanding of the needs and challenges of adolescents living with HIV in school settings, including barriers to HIV treatment adherence; mapping barriers to addressing HIV stigma within schools			Contact with adolescents living with HIV (presentations and testimonials from adolescents living with HIV)		Drivers and facilitators of stigma		Cognitive empathy; parasocial learning; skill building
Evaluating options for action planning for change; task analysis and developing an action plan; identification of stakeholders and resources to support initiatives to reduce stigma and support adolescents living with HIV in schools			Participatory learning through breakout sessions to brainstorm and develop actions plans for their schools		Future actions to support adolescents living with HIV; sustainable programs and policies to support adolescents living with HIV		Social learning theory: modeling; efficacy; empowerment through skill building
**Booster sessions**							
	Review of ongoing programs to support adolescents living with HIV in school setting (successes, challenges, and alternative strategies)	Empowerment; peer-to-peer learning		Institutionalizing change; knowledge generation and transfer		Social learning theory: modeling; efficacy; empowerment through skill building	

Previous studies have shown that direct or indirect contact (eg, digital film presentations) with stigmatized groups results in broader and more enduring reductions in stigma [152-154]. Thus, we will use expert testimonials from adolescents living with HIV as direct contact opportunities for the educators to hear their personal experiences in dealing with stigma and to *normalize* adolescents living with HIV as human beings; hence, fostering acceptance and empathy for adolescents living with HIV. Open discussions will provide a safe place for educators to express their views and opinions of adolescents living with HIV, as well as explore strategies, resources, and barriers to support adolescents living with HIV within schools. This strategy of actively engaging educators in examining their biases and developing supportive strategies for adolescents living with HIV within their settings is consistent with the principles of empowerment that build a sense of ownership. Participants will then act as change agents within their schools by implementing activities that address the needs of adolescents living with HIV. To facilitate context-specific discussions, we will conduct quarterly visits (at least one visit per academic term) to individual intervention schools in between the workshops to establish how educators are supporting adolescents living with HIV within their schools and offer additional services (eg, training) based on the requests from the schools.

### Ethics Approval and Consent

The research staff will obtain written informed consent and assent from the adult caregivers and children, respectively, before study enrollment. The consenting process for adults and children will be performed separately to avoid coercion. During face-to-face meetings, the adolescent’s primary caregiver will read and sign a standard consent form. In doing so, caregivers will be consenting to participation for themselves and assenting to the participation of their adolescents. Adolescents will sign an assent form that will be read aloud verbatim. If either the adolescent or caregiver refuses to participate, they will not be enrolled. According to the Uganda Law, emancipated minors, defined as persons aged ≤18 years who are pregnant, married, have a child, or are self-sufficient, will be allowed to consent on their own. Both consent and assent forms will be translated into Luganda (the most widely spoken local language in the study region) and back translated to English to ensure consistency. Both the assent and consent processes will be conducted verbally in Luganda, given that some caregivers and adolescents were illiterate. The study team will receive training on Good Clinical Practices so that sensitive research activities can be handled appropriately. In addition, all interviewers have completed the Collaborative Institutional Training Initiative certificate and National Institutes of Health certificate to safeguard the research participants.

We have obtained approval for the study procedures from the institutional review boards (IRBs) at the University of Washington in St. Louis, Missouri (IRB ID 202201128) and University of Michigan (HUM00211945) and from the in-country local IRBs in Uganda: Uganda Virus Research Institute (GC/127/867) and Uganda National Council of Science and Technology (SS1166ES).

The study has been registered with ClinicalTrials.gov (NCT05307250), as of April 1, 2022. The dissemination of the main study findings is targeted for 2025.

### Measures

As shown in Figure 1, assessment will be conducted at baseline and at 12-, 24-, and 36-month follow-ups. All assessments, each lasting approximately 60 minutes, will take place at the clinic during school breaks. Although all the adolescents living with HIV will be attending school and expect to be English-speaking (the instructional language in all Ugandan schools), assessments will be conducted in English or Luganda (the local language) depending on the English proficiency of the participants. All the interviewers will be fluent in English and Luganda. The questions will be translated from English to Luganda and back translated by a certified translator from a local university (Department of Languages) following standard procedures. The research team members who are fluent in Luganda and English will crosscheck all translated assessments. All the interviewers will receive highly structured and intensive training. Assessments will be conducted using standardized measures adapted from previous studies conducted in Uganda [104,133,141]. Any measures that have not been used will be pretested and made culturally appropriate to the Ugandan context. For questions measuring sensitive behaviors (eg, adherence), we will use audio computer-assisted self-interviews, where the participant takes the survey herself on a mini laptop. Nonsensitive questions will be administered by the interviewer. For the biological assay, blood specimens for HIV VL testing will be collected at baseline and 12, 24, and 36 months after the intervention. In accordance with the Abbott platform, VL will be dichotomized into undetectable (<40 copies/ml) and detectable (≥40 copies/ml) levels.

### Qualitative Component

Semistructured in-depth interviews will be conducted at baseline and at 12-, 24-, and 36-month follow-ups with adolescents living with HIV and their caregivers (n=40 dyads) in the 2 intervention arms. Baseline interviews will focus on the following aspects: (1) participants’ experience of decision-making (eg, costs, benefits, barriers, and facilitators) associated with HIV treatment adherence and (2) HIV stigma and its perceived impact on their lives. Follow-up interviews will unpack the longer-term impact, including experiences of stigma and key multilevel factors affecting HIV treatment–related behaviors of the participants after the intervention. Specifically, in addition to the baseline interview topics, 12-month interviews will examine the following: (1) experiences of the participants with their respective intervention components (ie, MFG-HIVSR, FEE, and GED-HIVSR), including perceived benefits and key multilevel (individual, family, school, contextual, and programmatic) influences that affect their participation and (2) intervention sustainability. In addition to topics explored at baseline (HIV stigma and decision-making on HIV treatment), the follow-up interviews will explore the sustained impact of the intervention to examine changes over time in HIV stigma and decision-making associated with treatment adherence and the sustained impact of the intervention over time.

In addition, educators (n=20) will be interviewed at baseline and at follow-up (12, 24, and 36 months). Baseline interviews will focus on their attitudes toward adolescents living with HIV and how HIV stigma manifests within their school context. Follow-up interviews, in addition to topics covered during baseline, will explore educators’ experiences with the training and resulting programs implemented within their school, facilitators and barriers to program implementation, recommendations, and sustainability. A purposive criterion sampling strategy [155] will be used to select adolescents living with HIV and their caregivers. adolescents living with HIV who score in the highest and lowest quartiles of internalized stigma at baseline (to be identified using the HIV stigma mechanism scale), and 20 participants (10 from each quartile) and their caregivers from each treatment condition will be randomly selected (n=40 dyads; these numbers will be sufficient for theoretical saturation) [156-158] and interviewed. This sampling method will ensure that participants with varying experiences are represented and will allow us to identify common patterns and variations across participants’ experiences. In addition, 20 educators across the 2 treatment arms will be randomly selected for interviews. Interviews will be conducted in English or Luganda, based on the participants’ preferences. The questions will be translated (English to Luganda) and back translated by research assistants, and then reviewed by 2 proficient team members (MM and PN). Each interview will last approximately 60 minutes and will be audiotaped. The same participants will be interviewed at each time point.

### Data Analysis

#### Primary Analyses for Aim 1

To examine the effect of M-Suubi on HIV viral suppression, we hypothesize the following:

- H1a: MFG-HIVSR plus FEE will have higher odds of viral suppression than control participants (bolstered usual care).
- H1b: MFG-HIVSR plus FEE plus GED-HIVSR will have higher odds of viral suppression than control participants.
- H1c: MFG-HIVSR plus FEE plus GED-HIVSR will have higher odds of viral suppression than MFG-HIVSR plus FEE.

To test these 3 hypotheses, we will fit a 3-level generalized linear mixed model (LMM) with fixed effects for the study arm, time, and their interaction. Our analysis will follow an intent-to-treat approach, such that all participants are included in the analyses, irrespective of whether they have complete or incomplete outcome data. Maximum likelihood (ML) and multiple imputation (MI) procedures will be used to address missing data with sensitivity analyses. Sensitivity analyses will be performed using pattern-based MI to examine the robustness of the results under different missing data assumptions. We will use random intercepts for school/clinic ID to account for clustering of persons within schools and their affiliated clinics and include random intercepts, random slopes, and their covariance for person ID to account for clustering of repeated measurements within persons. Reflecting the binary HIV viral suppression outcome, a binomial distribution and log link will be used to fit a log-binomial model to estimate the relative risks. If the log-binomial model does not converge, we will substitute a Poisson model with robust SEs [159,160]. To maximize rigor, quasi-likelihood methods will not be used. Instead, maximum likelihood estimation via adaptive Gaussian quadrature with 15 integration points will be used to ensure stable solutions [161]. To test hypotheses H1a to H1c, we will perform 3 time-averaged comparisons of repeatedly measured observations across study arms to examine the intervention effects over the duration of the study period. As all possible comparisons among the 3 study arms will be evaluated, the α will be set at .05/3=.017 for each of these 3 planned comparisons. Any additional post hoc comparisons (eg, paired comparisons of groups at each time point) will maintain a nominal α of .05 using simulation-based step-down multiple comparison methods [114]. Our team has considerable experience fitting 3-level generalized LMMs to analyze data from our cluster randomized asset-based intervention trials [113,162].

#### Primary Analyses for Aim 2

To examine the effect of M-Suubi on HIV stigma, we hypothesize the following:

1. H2a: MFG-HIVSR plus FEE will have lower mean HIV stigma than control participants (bolstered usual care).
2. H2b: MFG-HIVSR plus FEE plus GED-HIVSR will have lower mean HIV stigma than control participants.
3. H2c: MFG-HIVSR plus FEE plus GED-HIVSR will have lower mean HIV stigma than MFG-HIVSR plus FEE.

To test these hypotheses, we will fit LMMs using the same fixed effects (study arm, time, and study arm-by-time) and random effects for the school/clinic (random intercepts) and person levels (random intercepts, random slopes, and their covariance) as proposed in the H1 analyses described earlier. To test hypotheses H2a to H2c, we will perform 3 time-averaged comparisons of repeatedly measured observations of stigma across study arms to examine the intervention effects over the duration of the study. To maintain a nominal type 1 error rate of 5% across tests of H2a to H2c, α will be set at .05/3=.017 for each planned time-averaged comparison. Our analyses will follow an intent-to-treat approach, and ML and MI approaches will be used to address missing data (as described in aim 1 earlier). To maximize rigor, the assumptions of normality and constant variance of residuals for these continuous outcomes in LMMs will be evaluated by examining histograms of the residuals and scatter plots of predicted values-by-Cholesky-scaled residuals, respectively. Transformations of outcomes will be used as needed to improve data conformance with model assumptions. Inferences for models whose residual statistics still do not fully meet assumptions following transformations will be generated via robust heteroskedastic-consistent Huber-White “sandwich” variance estimators [163]. All analyses will include outlier and influential case screening via the computation of Cook *D*, DF β values, and likelihood displacement statistics. If outliers are found, the results will be reported with and without outliers included [164,165].

#### Randomization, Sample Size, and Power Analysis

We used NCSS Statistical Software Program PASS [166] to compute the minimum detectable effect size estimates for hypotheses H1a to H1c and H2a to H2c proposed to fulfill specific aims 1 and 2, respectively. For all power analyses, we assume power=0.80, α=.05/3=.017, and 4 repeated assessments from 714 participants conservatively assuming 15% attrition. Standardized minimum detectable effect sizes range from .26 to .35. Therefore, our study will have the power to detect small to medium effects for the proposed hypotheses

#### Aim 3: Evaluate the Cost-effectiveness of Each Intervention Condition

Following the standard practice of measuring the cost-effectiveness of interventions, we will measure costs on a per-person basis. The intervention costs will include all program costs incurred for running the GED-HIVSR and MFG-HIVSR plus FEE programs and not just the savings match of the youth development account. The research costs will not be included in this study. Data on the savings match costs will be readily available from the management information system. Data on the costs of other program elements will be drawn from the project administrative records collected throughout the intervention period. In the analyses, the costs from multiple years will be adjusted for inflation, depreciation, and discounting. The outcome analyses described earlier will be used to estimate the extent to which the *Combined Intervention (MFG-HIVSR plus FEE plus GED-HIVSR)* versus *MFG-HIVSR plus FEE alone* increased particular outcomes (eg, viral suppression). The per-person costs of *MFG-HIVSR plus FEE and GED-HIVSR* and *MFG-HIVSR plus FEE alone* will then be divided by the relevant effect sizes to produce estimates of cost-effectiveness. We will calculate CIs for point estimates using two methods: Monte Carlo [167] and bootstrap [168].

#### Aim 4: Qualitative Component Analysis

The interviews will be transcribed and uploaded to NVivo (version 12; QSR International) [169]. Data will be analyzed using a recurrent cross-sectional approach. Each wave of data will first be analyzed independently to understand experiences at each time point of data collection [170]. Analytic induction techniques [171] will be used for coding. Initially, 10 interview transcripts randomly selected across the 2 study groups will be read multiple times and independently coded by the team using sensitizing concepts to identify emergent themes (open coding) [172]. Broader themes will be divided into smaller, more specific units until no further subcategories are necessary. Analytic memos will be written to further develop categories, themes, and subthemes, and to integrate the ideas that emerge from the data [172,173]. Codes and the inclusion/exclusion criteria for assigning codes [174] will be discussed as a team to create the final codebook in NVivo. Each transcript will then be independently coded by 2 investigators using the codebook. Intercoder reliability will be established. A level of agreement ranging from 66% to 97% based on the level of coding indicates good reliability [155]. Disagreements will be resolved through team discussions. The secondary analysis will compare/contrast themes and categories within and across groups to identify similarities, differences, and relationships among the findings. Member checking, peer debriefing, and audit trails will be used to ensure rigor [158]. The data will be analyzed using both recurrent cross-sectional and trajectory approaches. After this initial analysis is completed, a second analysis will focus on the differences and similarities between the time points. Central themes from each wave of data collection will be compared using these 3 subsets of questions. The coded data will be organized into matrices with major themes (along the y-axis) and time points (along the x-axis) to explore how the data, in the existing thematic groupings, changed or did not change over time (eg, new concerns and change in priorities), as well as new major themes that emerge from one time point to another.

## Results

The M-Suubi study was initiated in September 2021. The first 6 months of this 5-year study were a preparation period for obtaining IRB approval, mobilizing financial institutions, and recruiting clinics and adolescents. Data collection commenced in April 2022, with screening and recruitment of study participants, as well as completion of baseline assessments. Implementation of the MFG-HIVSR, GED-HIVSR, and FEE components will follow after randomization of the study participants, and the intervention will be delivered over a period of 20 months. Follow-up assessments will be conducted at 12, 24, and 36 months after completion of the baseline assessments.

## Discussion

### Overview

To the best of our knowledge, this is the first study to evaluate a culturally acceptable multilevel intervention to reduce HIV stigma within homes and schools and to improve HIV treatment adherence among in-school adolescents living with HIV in Uganda. HIV stigma reduction interventions targeting adolescents living with HIV in boarding school sections are nonexistent, and multilevel interventions addressing intrapersonal, interpersonal, and institutional stigma are scarce. The MFG approach is culturally consistent with SSA’s collective approach of families raising children “together,” which strengthens its appeal to communities and its likelihood of success. The asset-savings–led approach has demonstrated efficacy in reducing HIV-risk behaviors among HIV-affected adolescents [78,104,105,131,175,176] and has improved ART adherence among adolescents living with HIV [26]. The focus on schools is consistent with the United Nations Educational, Scientific and Cultural Organization’s Good Policy and Practice on HIV in Schools report [176], which established a road map for supporting schools as caring contexts for children affected by HIV/AIDS. M-Suubi makes use of existing community institutions to deliver the intervention and builds local capacity, which will ensure an eventual scale-up. M-Suubi will provide much-needed evidence on effective strategies for reducing HIV stigma among school-attending adolescents living with HIV in Uganda. More importantly, this study will provide evidence on the effects of a multilevel intervention comprising of family-based economic empowerment and financial literacy combined with a school-based HIV stigma reduction intervention for educators. In so doing, it will enable an ecological assessment of the cascading effects of multilevel HIV stigma reduction strategies. In addition, the inclusion of educators as a target population will provide a unique opportunity to generate data on the prevalence and impact of HIV stigma among educators and effective intervention strategies to reduce HIV stigma within schools. To date, these data are nonexistent.

### Limitations

This study has some limitations. First, it targets adolescents living with HIV in southwestern Uganda, so the study findings may not be generalizable to adolescents living with HIV in Uganda or other high HIV burden countries in SSA. Second, the study focuses on adolescents living with HIV attending primary or secondary school. adolescents living with HIV in vocational schools and other nontraditional school settings are not included in the study, which may bias the generalization of the study findings. Nonetheless, this study uses a sound methodological approach, which will enhance the quality of data generated in this study. The study findings, if successful, would advance knowledge to bridge the existing gap in evidence-based scalable HIV stigma interventions for adolescents living with HIV in resource-limited settings such as Uganda.

## Appendix

Multimedia Appendix 1 Peer review report from the Risk, Prevention and Health Behavior Integrated Review Group - Center for Scientific Review (CSR) Special Emphasis Panel - (National Institutes of Health, USA).

## Abbreviations

ART: antiretroviral therapy
FEE: family economic empowerment
GED-HIVSR: group-based HIV stigma reduction for educators
IRB: institutional review board
LMM: linear mixed model
MFG: multiple family group
MFG-HIVSR: MFG HIV stigma reduction for educators
MI: maximum imputation
ML: maximum likelihood
SSA: sub-Saharan Africa
VL: viral load

## Data Availability

Data sets from this study will be available to researchers through the National Institutes of Health Central Data Repository.
