# Supplementary material for: A Multilevel Integrated Intervention to Reduce the Impact of HIV Stigma on HIV Treatment Outcomes Among Adolescents Living With HIV in Uganda: Protocol for a Randomized Controlled Trial
Source: JMIR Res Protoc. 2022 Oct 5;11(10):e40101. doi: 10.2196/40101 (PMC9582915; doi:10.2196/40101)
Supplement: Multimedia Appendix 1 [file resprot_v11i10e40101_app1.pdf]

SUMMARY STATEMENT
(Privileged Communication)

PROGRAM CONTACT:
Susannah Allison
240-627-3861
allisonsu@mail.nih.gov

Release Date: 04/08/2021
Revised Date:

Application Number: 1 R01 MH126892-01A1
Formerly: 1R01MH126892-01

Principal Investigators (Listed Alphabetically):

MUTUMBA, MASSY
SSEWAMALA, FRED M (Contact)

Applicant Organization: WASHINGTON UNIVERSITY

Review Group: ZRG1 RPHB-P (02)
Center for Scientific Review Special Emphasis Panel
Member Conflict: HIV/AIDS Related Behavioral Research
AIDS - EXP. REV.

Meeting Date: 03/30/2021
Council: MAY 2021
Requested Start: 07/01/2021

RFA/PA: PA20-144
PCC: 9A-ASPA

Project Title: M-Suubi: A Multi-level integrated intervention to reduce the impact of HIV stigma on HIV treatment outcomes among adolescents living with HIV in Uganda
SRG Action: Impact Score:31 Percentile:16 #
Next Steps: Visit https://grants.nih.gov/grants/next_steps.htm
Human Subjects: 30-Human subjects involved - Certified, no SRG concerns
Animal Subjects: 10-No live vertebrate animals involved for competing appl.
Gender: 1A-Both genders, scientifically acceptable
Minority: 5A-Only foreign subjects, scientifically acceptable
Age: 2A-Only Children, scientifically acceptable

Project Year	Direct Costs Requested	Estimated Total Cost
1	499,685	691,680
2	499,968	692,072
3	499,741	691,758
4	499,844	691,900
5	499,937	692,029
TOTAL	2,499,175	3,459,440

ADMINISTRATIVE BUDGET NOTE: The budget shown is the requested budget and has not been adjusted to reflect any recommendations made by reviewers. If an award is planned, the costs will be calculated by Institute grants management staff based on the recommendations outlined below in the COMMITTEE BUDGET RECOMMENDATIONS section.

1R01MH126892-01A1 SSEWAMALA, FRED

RESUME AND SUMMARY OF DISCUSSION: The overall goal of this application is to examine an evidence-informed HIV stigma mitigation intervention, called Multilevel Suubi (M-Suubi), that seeks to address multiple barriers to HIV treatment adherence and care engagement among adolescents living with HIV (ALHIV) attending boarding schools in Uganda. This is a significant project focusing on a highly vulnerable population of ALHIV who are negatively impacted by stigma and poverty as barriers to antiretroviral therapy (ART) adherence. If successful, the multilevel, combined intervention approach could simultaneously address these barriers, thus, mitigating a significant public health concern. The investigative team are recognized leaders in their fields, have a history of collaboration, and a successful track record of conducting clinical trials in Uganda. Innovation was acknowledged with the multi-level approach, which includes educators and family components, for addressing barriers to HIV treatment adherence. Strengths in the approach include a robust study design, appropriate measures, and a multi-level stigma reduction and economic empowerment HIV prevention intervention. However, concerns were raised with how the proposed intervention and subsequent analyses would disentangle the effects of the stigma mitigation component alone. Furthermore, the theoretical framework and stigma domains weren't entirely well-mapped to the intervention or analyses. The panel agreed the resubmission was modestly responsive to prior review. However, a remaining concern consisted of insufficient details with the qualitative analysis plan. These considerations did not significantly reduce the project's overall merit. Overall, this was a significant resubmission led by a strong study team, yielding a sound project with an overall high impact for HIV prevention among ALHIV.

DESCRIPTION (provided by applicant): In response to PA-20-144 calling for innovations in HIV prevention, testing, adherence and retention to optimize HIV prevention and care continuum outcomes, this proposal examines the impact and cost-effectiveness of an innovative multilevel intervention combining group-based HIV stigma reduction for Educators (GED-HIVSR) and Multiple Family Groups (MFGs) for HIV stigma reduction (MFG-HIVSR) with a family economic empowerment intervention on HIV treatment adherence and engagement in care among school-going adolescents living with HIV (ALHIV) in Uganda. Uganda is home to over 170,000 ALHIV. ALHIV have lower levels of ART adherence, low rates of viral suppression, and high attrition from HIV care. ALHIV in boarding schools are more disadvantaged and have lower levels of HIV treatment adherence. HIV stigma, poverty (including food insecurity), and poor mental health are the most potent barriers to ART adherence in Uganda and sub-Saharan Africa (SSA). The school social context is very disadvantageous to ALHIV due to lack the family support that typically facilitates treatment adherence; lack of privacy coupled with high levels of HIV stigma; and poverty related food insecurity, manifesting as lack of food to accompany medication. While several interventions have shown promise in improving HIV treatment adherence among ALHIV, they mostly focus on ALHIV commuting daily from home. Yet, the majority of school-going children in Uganda, and many SSA countries heavily impacted by HIV, spend their time in boarding sections. The lack of attention to this group undermines efforts to achieve the 95-95-95 targets in SSA. Building on our experience and evidence of effective HIV stigma reduction strategies; and guided by theory, we propose to examine an evidence-informed multilevel intervention called Multilevel Suubi (hope) intervention (hereafter, M-Suubi) among ALHIV attending boarding schools in Uganda. The study will be guided by four specific aims: Aim 1: Examine the impact of M-Suubi on HIV viral suppression (primary outcome); and adherence to HIV treatment (keeping appointments, pharmacy refills, pill counts), and retention in care (secondary outcome); Aim 2: Examine the effect of M-Suubi on HIV stigma (internalized, anticipated, and enacted), with secondary analyses to explore hypothesized mechanisms of change (e.g. depression) and intervention mediation; Aim 3: Assess the cost and cost-effectiveness of each intervention condition; and Aim 4: Qualitatively examine: a) participants' experiences with HIV stigma, HIV treatment adherence, and the intervention; and 2) educators' attitudes towards ALHIV and experiences with GED-HIVSR, and program/policy

implementation post-training. The study will enroll 840 ALHIV recruited from 42 schools in the greater Masaka region, heavily affected by HIV (prevalence 12% vs 7.3% national average). M-Suubi will be provided for 20 months, with assessments at baseline, 12, 24 and 36 months. Findings may inform combination intervention efforts to optimize HIV treatment outcomes and engagement in care among ALHIV.

PUBLIC HEALTH RELEVANCE: Aligned with NIH's strategic priority to support innovations in HIV prevention, testing, adherence and retention to optimize HIV care continuum outcomes, the overall goal of the proposed study is to examine the impact and cost-effectiveness of an innovative multilevel intervention on HIV treatment adherence and engagement in care outcomes among school-going adolescents living with HIV (ALHIV) in Uganda. This R01 will combine group- based HIV stigma reduction for educators and Multiple Family Groups for HIV stigma reduction with a family economic empowerment intervention to address stigma and economic barriers associated with treatment outcomes and engagement in care. Findings may inform combination intervention efforts to optimize HIV treatment outcomes among ALHIV in Uganda and other countries in SSA.

CRITIQUE 1

Significance: 2
Investigator(s): 2
Innovation: 2
Approach: 5
Environment: 1

Overall Impact: This is a responsive resubmission application for a three-arm randomized control trial of a multi-level intervention to promote HIV treatment adherence and care engagement among adolescents living with HIV ALHIV (n=480) who attend boarding schools (n=42) in Uganda. The study is designed to test the effects of school only vs. school plus family-level interventions, with a focus on addressing to key barriers to ART adherence: stigma and poverty. The study is highly significant in that it addresses the context of ALHIV in boarding schools. The proposed intervention integrates attention to stigma and poverty at the individual, family and school and multiple types of participants (ALHIV, parents and teachers/school admin/staff). The resubmission includes more detail and clarity about key aspects of the intervention, in particular how it will address both poverty and stigma, and also addressed design concerns raised by previous reviewers. They also highlighted the preliminary work done by this team to inform the current proposal. Ongoing weaknesses are the lack of innovation in the approach to stigma reduction at both school and family levels, lack of precision in how the intervention will address specific forms of stigma and measure these specific forms, lack of attention to how to manage and analyze longitudinal qualitative data, and lack of discussion of sustainability of the economic intervention.

1. Significance:

Strengths

- Focus on a clearly defined vulnerable group (adolescents in boarding schools) with opportunity to generate transferable findings given that boarding schools for adolescents are commonly used across many settings.
- Well-developed argument for the negative impacts of poverty and stigma among ALHIV in boarding schools.

Weaknesses

- Not clear how anticipated stigma is conceptualized as “external” when, like internalized stigma, it is experienced by the individual, rather than enacted upon the individual by someone else.

2. Investigator(s):

Strengths

- Thoughtful MPI (Ssewamala and Mutumba) approach that will both maximize existing capacity and provide opportunity for junior investigator mentorship and leadership development.
- Existing collaborations (grants and papers) between members of the research team.
- Strong record of retention in past research in this setting encourages confidence in proposed approach to recruit and retain.

Weaknesses

- Co-I Sensoy Bahar has extensive qualitative experience and has worked with the proposed team but it is not clear if she has experience with *longitudinal* qualitative research in the context of program evaluation.

3. Innovation:

Strengths

- Integrating stigma reduction with poverty reduction.
- Asset orientation of the intervention is an innovative approach for a stigma reduction intervention.

Weaknesses

- The actual content and proposed techniques proposed within the intervention components are not highly innovative, in particular with regard to the stigma content with teachers and families is not innovative.
- It is not clear how the intervention assessment will be able to capture “cascading effects of multi-level stigma”.

4. Approach:

Strengths

- Strong, robust design that will allow for determining the relative advantage of intervening at both family and school levels.
- School level randomization is reasonable for intervention.
- Use of unconditional matching maximizes ALHIV autonomy and flexibility.
- 36-months of follow-up.

Weaknesses

- GED-HIVSR content: The domains are not well defined and do not map onto the theoretical framework, which specifies attention to specific types and forms of stigma that are not reflected in the domains. There is also disconnect between the domains and the proposed topical content whereby the content appears to be broader than the domain.

- There is also lack of reflection of forms of stigma (internalized, enacted, anticipated) highlighted in the theoretical framework and the evaluation measure, which will limit the ability of the study to assess stigma in a holistic and specific way. This is a major concern given that the study is focused on stigma. In the analyses, the hypotheses just refer to "HIV stigma", without any reference to what form/domain.
- The resubmission application includes more detail about one specific approach to qualitative analysis (trajectories), there is still insufficient detail in the proposal about the process of iteratively collecting qualitative data over time, how the data will be managed, and the distinct analytic techniques, beyond coding, that will be used to maximize insights that can be obtained from this longitudinal qualitative data. As described, it is not clear that Aim 4 will be used to its full advantage.
- While the proposal highlights the importance of capacity building and engagement with local community institutions, there is no discussion of the sustainability of the poverty reduction/matching intervention.

5. Environment:

Strengths

- US institutions and Ugandan sites offer outstanding environments.

Weaknesses

- No concerns other than the potential for over saturation of HIV research in this setting.

Study Timeline:

Strengths

- Overall, the timeline is reasonable.

Weaknesses

- The intervention implementation is quite condensed and short, even with the boosters, though this may be appropriate for the target population (especially, teachers).

Protections for Human Subjects:

Acceptable Risks and/or Adequate Protections

Data and Safety Monitoring Plan (Applicable for Clinical Trials Only):

Acceptable

Inclusion Plans:

- Sex/Gender: Distribution justified scientifically
- Race/Ethnicity: Distribution justified scientifically
- For NIH-Defined Phase III trials, Plans for valid design and analysis: Not applicable
- Inclusion/Exclusion Based on Age: Distribution justified scientifically

Vertebrate Animals:

Not Applicable (No Vertebrate Animals)

Biohazards:

Not Applicable (No Biohazards)

Resubmission:

- Response in terms of clarifying previous reviewer doubts/questions. No fundamental changes were made but this is consistent with the feedback provided. The longitudinal qualitative component still feels underdeveloped and the connection between intervention framework actual intervention activities and measures

Resource Sharing Plans:

Acceptable

Budget and Period of Support:

Recommend as Requested

CRITIQUE 2

Significance: 3
Investigator(s): 3
Innovation: 3
Approach: 4
Environment: 1

Overall Impact: This application seeks to test a combination intervention approach to improve medication adherence among adolescents living with HIV in Uganda. The study team is comprised of investigators with substantial track records of successfully conducting clinical trials in Uganda. A 3-arm RCT will compare standard of care to an arm with stigma reduction intervention and to an arm with stigma reduction and economic empowerment in the form of matched investments. The primary study outcome is viral suppression. Changes in stigma ratings will be assessed, and cost-effectiveness analyses conducted. Enthusiasm for the approach is limited in part due to the most novel components of the study, stigma reduction components, being not sufficiently detailed and not previously developed. There is no pilot test for these new intervention components, and also, they are not sufficiently integrated with the theoretical framework of the study. Nonetheless, this is a strong application on an important topic from an excellent team.

1. Significance:

Strengths

- The project addresses a highly impact population and justifies the importance of addressing the unique population of Uganda adolescents in boarding schools.
- The combination HIV prevention approach is a strength, and the addition of economic support to stigma-based approaches that have yielded limited efficacy alone in the past is promising.

- The application provides a compelling picture of the need for and complement of these disparate approaches.
- Targeting adherence may have both health implications for the individual and preventive benefits for the population.

Weaknesses

- A number of the adherence challenges raised in the section, such as lack of privacy in boarding school settings and stigma from other students, are not addressable by the proposed intervention.

2. Investigator(s):

Strengths

- PI Ssewamala has demonstrated excellence in conducting RCT and related research in the proposed setting, and MPI Mutumba complements this expertise.
- Highly experienced overall team, with a demonstrated track record of collaboration including successfully completed trials and disseminated results.

Weaknesses

- Lack of investigators highly expert in developing and implementing stigma reduction interventions is a minor limitation.

3. Innovation:

Strengths

- Uses a novel, multilevel approach to reduce stigma.
- The combination of a clinical trial with longitudinal qualitative components provides an opportunity to delve into some of the underlying issues regarding stigma.
- Addressing stigma among educators is a novel approach.
- Combining stigma reduction with economic empowerment is novel and interesting, having the potential for a synergistic effect.

Weaknesses

- The study design, in allocating interventions, would not allow for the disaggregation of the effect of an intervention previously demonstrated to be effective (economic empowerment) from a stigma mitigation intervention (family-based) that lacks evidence of efficacy. So, the synergy could not be measured.
- The broader scientific generalizability and potential theoretical knowledge gained, beyond efficacy of the specific intervention, is not substantially addressed.

4. Approach:

Strengths

- School-level randomization is rigorous and an appropriate design.
- The use of biomarkers for outcome measure is a strength, and analyses of primary outcome are well-specified and appropriate.

- The secondary measurement of stigma outcomes is also a strength, and the mediation that assesses whether changes in stigma are along the causal pathway, using SEM, is a strength.
- Longitudinal qualitative exploration regarding the stigma program will yield useful data, informing interpretations of study results.
- Cost-effectiveness approach will inform long-term intervention use and prioritization.

Weaknesses

- The study does not seem to have the intervention package ready for the trial, yet doesn't propose any developmental or pilot work. The stigma intervention for educators (arm 2) and the stigma additions to the economic intervention (arm 3) will need to be developed. That development plan is lacking. Furthermore, these new interventions should have some kind of pilot testing prior to the launch for such a substantial clinical trial.
- It is unclear how some of the past curricula, Suubi-MAKA and Suubi+Adherence, will be incorporated into the intervention.
- The cost-effectiveness approach lacks detail. For instance, it is unclear if indirect costs will be considered. Moreover, it is unclear if the approach will use a metric such as disability adjusted life year that could be used to compare cost-effectiveness results beyond the horizon of this particular study, to facilitate intervention prioritization.
- Unable to find prior studies that make the multifamily group program that addresses stigma an evidence-based intervention, or even one that has been used before. Citations regarding intervention content for this component lead to economic empowerment literature, not stigma or family stigma support literature.
- The stigma intervention for families (and educators) is not sufficiently detailed, with little information regarding the content and how it will connect to theory. In particular, how does the intervention content address theoretical domains of enacted, anticipated, and internal stigma?
- The qualitative component lacks a bit of detail regarding the exact domains to be explored in the interviews, and how these will be connected to critical questions regarding the intervention and its context and effects.

5. Environment:

Strengths

- Excellent research environment, with documented success at each site.
- Support from local organizations and the community of the overall research team is a strength.

Weaknesses

- None of note.

Study Timeline:

Strengths

- The timeline is generally appropriate.

Weaknesses

- It does not appear that the intervention package, particularly stigma-related components, have been designed and are ready for immediate launch; time to develop these materials and gain feedback on them should be added to the study timeline.

Protections for Human Subjects:

Acceptable Risks and/or Adequate Protections

- The study minimizes risk to participants appropriately.

Data and Safety Monitoring Plan (Applicable for Clinical Trials Only):

Acceptable

Inclusion Plans:

- Sex/Gender: Distribution justified scientifically
- Race/Ethnicity: Distribution justified scientifically
- For NIH-Defined Phase III trials, Plans for valid design and analysis: Not applicable
- Inclusion/Exclusion Based on Age: Distribution justified scientifically

Vertebrate Animals:

Not Applicable (No Vertebrate Animals)

Biohazards:

Not Applicable (No Biohazards)

Resubmission:

- The resubmission addresses most concerns held by previous reviewers, in particular the readiness of the contact PI. It does not fully address concerns regarding the limited integration of theory into the intervention and does not address concerns regarding the need to provide additional detail regarding the intervention approach for the non-economic intervention components.

Resource Sharing Plans:

Acceptable

Budget and Period of Support:

Recommend as Requested

CRITIQUE 3

Significance: 1
Investigator(s): 1

Innovation: 2
Approach: 2
Environment: 1

Overall Impact: This is a proposal to conduct a well-designed, multi-level intervention to reduce the impact of HIV stigma and poverty on HIV outcomes via mental health-related pathways among adolescents living with HIV (ALHIV). There is a tremendous need for HIV care outcomes intervention among ALHIV, who number 170,000 in Uganda, where the study is set. The proposed study will focus on children in boarding schools and will include emancipated minors. The investigators propose to expand an existing intervention to include the school (educators) and family levels and test it via 3-arm cluster randomized trial implemented across 42 secondary schools with a boarding component (n= 14 schools, n=280 students per study arm) in areas heavily affected by HIV. This resubmission application is significantly strengthened by a focus on poverty and mental health as causes and mediators respectively of adverse outcomes, viral suppression (primary outcome); and adherence to treatment (operationalized as keeping appointments, pharmacy refills, pill counts), and finally retention in care (secondary outcome). The applicants have addressed the major critiques from the first review adding more time for the investigators and creating an mPI plan and study. The rationale and scientific premise are very strong and the study design is sound, analysis includes generalized linear mixed (multilevel) models, and powered to detect a minimum 15% (raw) difference over 36 months of follow-up, which is an especially long period. The proposal is innovative as it proposes test a theory-based intervention via a challenging multilevel study design, but one that is needed to address the various sources of stigma and influences on HIV care related outcomes. The design is thoughtful and includes a qualitative component. The proposal has the potential for significant impact.

1. Significance:

Strengths

- The need to reduce stigma and support improvements in HIV care related outcomes (viral suppression) among this population is immense.

Weaknesses

- None noted by reviewer.

2. Investigator(s):

Strengths

- The investigative team is excellent with expertise in the areas required to successfully execute the study.
- The concerns related to effort and team organization of prior reviews have been addressed.

Weaknesses

- None noted by reviewer.

3. Innovation:

Strengths

- Testing a multi-level intervention that includes education and family (caregiver) components is very innovative and challenging.

Weaknesses

- None noted by reviewer.

4. Approach:

Strengths

- The study approach is sound and very clearly described.

Weaknesses

- Issues related to the economic empowerment component that could affect retention are not addressed.
- The stigma intervention could have been elaborated on more, although the framework and sessions are outlined.

5. Environment:

Strengths

- Excellent environment for the study.

Weaknesses

- None noted by reviewer.

Study Timeline:

Strengths

- Detailed and reasonable if ambitious timeline for the study.
- Information on recruitment and retention is provided.

Weaknesses

- None noted by reviewer.

Protections for Human Subjects:

Acceptable Risks and/or Adequate Protections

Data and Safety Monitoring Plan (Applicable for Clinical Trials Only):

Acceptable

Inclusion Plans:

- Sex/Gender: Distribution justified scientifically
- Race/Ethnicity: Distribution justified scientifically
- For NIH-Defined Phase III trials, Plans for valid design and analysis: Not applicable
- Inclusion/Exclusion Based on Age: Distribution justified scientifically

Vertebrate Animals:

Not Applicable (No Vertebrate Animals)

Biohazards:
Not Applicable (No Biohazards)

Resubmission:

- Acceptable

Resource Sharing Plans:
Acceptable

Budget and Period of Support:
Recommend as Requested

THE FOLLOWING SECTIONS WERE PREPARED BY THE SCIENTIFIC REVIEW OFFICER TO SUMMARIZE THE OUTCOME OF DISCUSSIONS OF THE REVIEW COMMITTEE, OR REVIEWERS' WRITTEN CRITIQUES, ON THE FOLLOWING ISSUES:

PROTECTION OF HUMAN SUBJECTS: ACCEPTABLE

INCLUSION OF WOMEN PLAN: ACCEPTABLE

INCLUSION OF MINORITIES PLAN: ACCEPTABLE

INCLUSION ACROSS THE LIFESPAN: ACCEPTABLE

COMMITTEE BUDGET RECOMMENDATIONS: The budget was recommended as requested.

Footnotes for 1 R01 MH126892-01A1; PI Name: SSEWAMALA, FRED M

Ad hoc or special section application percentiled against "Total CSR" base.

NIH has modified its policy regarding the receipt of resubmissions (amended applications).See Guide Notice NOT-OD-18-197 at https://grants.nih.gov/grants/guide/notice-files/NOT-OD-18-197.html. The impact/priority score is calculated after discussion of an application by averaging the overall scores (1-9) given by all voting reviewers on the committee and multiplying by 10. The criterion scores are submitted prior to the meeting by the individual reviewers assigned to an application, and are not discussed specifically at the review meeting or calculated into the overall impact score. Some applications also receive a percentile ranking. For details on the review process, see http://grants.nih.gov/grants/peer_review_process.htm#scoring.

MEETING ROSTER

The roster for this review meeting is displayed as an aggregated roster that includes reviewers from multiple CSR Special Emphasis Panels of the Risk, Prevention and Health Behavior Integrated Review Group
for the 2021/05 council round.
This roster for CSR is available at:
http://public.era.nih.gov/pubroster/Reports?DOCTYPE=SEP&DESFORMAT=PDF&AGENDA_SEQ_NUM_P=416254
